# Microscopy of the Dentinal Tubuli

**Published:** 1869-08

**Authors:** S. P. Cutler

**Affiliations:** Holly Springs, Miss.


### MICROSCOPY OF THE DENTINAL TUBULI.

BY S. P. CUTLER, M. D , A. E. G., D. D. S., HOLLY SPRINGS, MISS.

It might be supposed that the above subject is already overdone, hackneyed, threadbare and seedy. On the contrary, this subject is not even a settled question as yet; still discrepancies of opinion exist in relation to the exact anatomy and functions of these structures. Several years ago when I first published some articles on the above subject, the microscopists of Europe had adopted the views of Kölliker and others, and a large majority in this country who had written any thing on the subject at all. It is well known that Kölliker had conceived the idea of a fluid filling and occupying the tubuli and vicariously fulfilling the functions of sensorial nerves, besides that of nutrition. Now the latter function may be carried on beyond the terminal branches of nerves, as in cartilages, tendons and true bone structure, but there can not be shown an instance where true sensibility can be manifested without the influence of nerves per se. The very idea then, of supposing the sensibility of dentine depends on a fluid alone, to me seemed preposterous in the extreme; hence, I went to work in earnest to upset what seemed to me to be so great an absurdity. When I announced that a large molar tooth had from ten to twenty or more millions of nerve filaments, the very idea met with ridicule. I now propose to let any person make their own calculations, and see how far from actual truth I may have been led. Let any competent microscopist prepare a specimen of dentine, just under the enamel, where all the coronal or distal tubuli perforate the specimen, and measure their distances apart or see how many there are to the line, then square the number, he will find there is about 3,000 to the lineal inch; now let him square that and see what the result will be; he will find he has 9,000,000, which would be a small average for molar surfaces, even after the cement and enamel is removed, and in an extreme case two superficial square inches may be calculated on. In the above calculation I have not taken into consideration the incalculable numbers of minute branches found in the roots of teeth, running out laterally on all sides from the tubuli throughout their whole course from nerve cavity to the coronal branches. When these branches are all taken into account, it will be almost impossible to conceive of their numbers. In some roots these lateral branches are found to be much more numerous than in others. These lateral branchings are never found in the tubuli that lead up to the enamel, but may be found in the balance, though not always. In the roots the tubuli penetrate and reach the surface of cement through the anas-tomosis of the tubuli and canaliculi, this fact being proven by the sensibility of denuded or naked roots from absorption of alveolus and gums. In such cases the root surface sometimes becomes exceedingly sensitive to the touch of the finger-nail or brush. All Dentists have witnessed this fact, even in cases where almost the entire root has been exposed for years, showing the great tenacity of life of the nerve fibrils in dentine and cement. This fact of itself would be sufficient, if no other proof existed, to establish, beyond doubt, the existence of true nerve fibrils in the tubuli, at least in the roots of teeth. The proof of the existence of nerve fibrils in tubuli of crown present, if any odds, still stronger proofs. First, take the file and cut down through the enamel to the dentine; in many cases pain will be felt, not by filing down on to tubuli filled with water or any other fluid, but by wounding of true sensorial nerve filaments by the thousands, each and all contributing their share to the aggregate amount of pain produced. Again, in cases of sensitive decays in teeth, when the excavator is raked across the floor of the cavity, sharp, intolerable pain will be felt, causing nervous patients to writhe. Could such intense suffering be caused by opening tubes filled only with fluid? What a preposterous idea? Who can credit this? Now, for the sake of argument, let us admit that the tubuli are occupied alone by fluid; when decay reaches and perforates these tubes, what prevents the fluid from escaping from the tubes into the cavity of decay? There is no good reason to suppose that such would not be the case, then what would become of the medium of sensibility? Can any one who has paid close attention to the study of the sensibility of the teeth, entertain any such opinion, especially if he has made minute microscopic investigations of the teeth? When a great man announces a fact or hypothesis, all are ready to adopt his opinion without further inquiry. This seems to be the case in relation to the fluid hypothesis, as above stated. Nutrition, by osmotic force, may take place beyond the limits of nerve fibrils, but never sensibility or motion.

The teeth, like the dermis, combines the three great systems, the dermal, neural and hemal; the enamel representing the epidermis, the dentine the dermis, the blood vessels of the pulp representing the hemal, and the mucus of pulp and fluids the neural. The basement membrane encloses the hemal and neural system in the teeth, but leaves the intertubular spaces, mostly composed of lime, outside of this basement membrane. This membrane may be supposed to line the pulp canal and the tubuli throughout their entire extent, and cover the ends of fibrils at their terminations under the base of the enamel and surface of cement, thereby entirely enclosing the fibrils throughout their entire extent, and each one separately, after leaving pulp cavity and entering the tubes. In the case of the teeth the basement membrane is projected far beyond the hemal. This, however, is the case every where throughout the system, though to a limited extent, except in a few instances, the corneal coat of the eye being most striking; also, crystalline lens. The enamel may be regarded as exuvious, only being so hard that desquamation does not take place is not thrown off like the epidermis, and constantly rebuilt by the basement at its surface by actual evolution of new cells from the protoplasm. The dentine can not be supposed to belong to the osseous system, as it is not enclosed within this basement system, as that membrane is supposed to be a closed sack, without being perforated or penetrated any where. The osseous system is included within that shut sack. In other words, the animal proper dwells within this sack, and all nutritive plasma and oxygen on the one hand penetrate this sack from without, and carbonic acid, water, ammonia and other waste matters pass out through this membrane by what is perhaps improperly termed *osmosis*, *exosmosis* and *endosmosis*. These agents and elements of decay and reproduction all pass this closed sack without any open doors or windows, the membrane permitting them to pass through as gases and liquid, in a normal condition only. How they find their way through it is not necessary to discuss in this paper. As to the nutrition of tubular filaments occupying the dentinal tubes, they may be presumed to derive their supply from the pulp, first passing out around the fibrils and within the delicate neurilemma supposed to enclose the filaments, or they may draw their entire nutrition from the liquor sanguinis found to exist outside of pulp in pulp cavity, then passing through the tubes outside of fibrils, where there is sufficient space for such purpose. This fluid, in this case, would be carried along the inner walls of tubes, not by osmosis, but by capillary force or attraction, and again into the substance of the filament itself, throughout their entire course, by osmosis through the membrane or neurilemma. Now, if this membrane be regarded as the basement membrane, and not that lining the bony walls of pulp cavity and tubuli; then in this case the nutritive plasma must first pass outside of the basement membrane, and then in again; after entering the tubes which would constitute an anomaly in this instance and perhaps peculiar to the teeth alone. Let it be as it may, there is found to exist a fluid in pulp cavity and in tubuli, outside of nerve fibril, which does perhaps, in some cases of decay at least, if not all, pour out though in exceedingly small quantities, a fluid or moisture. Even after a tooth is filled there may be a certain amount of fluid coming to the surface of the decay around the filling. In cases of an acid diathesis, especially in young subjects, sufficient may accumulate at the surface of cavity and around the filling to set up a softening and ultimately dropping of filling, from the corroding effects of the acid, and the tooth is rapidly destroyed, unless treated and refilled. I am confident, I have had such cases in my practice. In some cases there might be another cause assigned for the presence of this acid. The nerve filaments running up to the bottom of the cavity may ultimately decompose or loose their vitality after a time, by the impressions of heat and cold through the conducting agency of the filling, and by their decomposition sufficient acid might be generated to ultimately soften the dentine at the bottom of the cavity, and subsequently loosen the filling so as to let it drop out, at the same time most of the nerve filaments on the sides of the cavity retaining their vitality and the pulp itself remain comparatively healthy. All unfavorable forms of decay, especially the white, should receive treatment, or at least an application of creosote and tannin, and retention for a sufficient length of time to allow capillary absorption into tubuli around fibrils. Styptic colloid may be used in some cases with decided advantage, allowing a pellicle to remain in the bottom of cavity as a non-conductor, and fill in the usual way. Returning to the fluid hypothesis: How could it be possible for a fluid, supposing the tubes contained nothing else, to be put in motion so as to cause pain by impinging on the pulp in its cavity by simply raking an excavator across the cavity, so as to wound the tubuli. The sharp, thin edge cutting across could not act like a piston in a force pump, so as to force the fluid rapidly through the tubes. Supposing such a thing possible and that such tubuli had a small piston forced into them through the decay what would be the result? The amount in these minute tubes, when forced into the pulp cavity, would act precisely like a hydrostatic press; the power would be very great, provided there was sufficient fluid in the tubes to forcibly condense the fluid already in the pulp cavity. The power in the supposed case would be multiplied in proportion to the diameter of the aggregate tubuli, multiplied into the superfices of pulp chamber; that is, the power exerted on the pistons applied to tubes. But no such thing can be supposed, as an excavator can not be supposed to produce any such result. Even admitting, for sake of argument, that such be the case, the pressure would be equal on all sides throughout the pulp cavity, and the only effect produced on the pulp would be simple pressure alone, and who could say that even acute pain would be the result of such pressure. No such condition of things can possibly exist. I contend that a fluid like liquor sanguinis could not be forced rapidly through tubes as small as tubuli; the largest at the nerve cavity being 1-100000 of an inch, and just under the enamel 5 or 6 times smaller, where generally the greatest sensibility is experienced. The pain is felt the very instant the touch is made with the rapidity of thought. The only mode by which a fluid can be made to pass through such small tubes, through such an unyielding substance as the dentine, is by capillary attraction or force, which requires a given time, and perhaps no degree of force could in any way accelerate the motion of the fluid in such tubes. Need I argue further? Is not the proof convincing to every one?

Is cement true bone? I maintain it is something more, as when the surface of a root of tooth has been denuded and apparently dry for a length of time, there is frequently felt great sensibility. No such sensibility is felt where true bone has been denuded, as the canaliculi and lacunæ of bone do not contain nerve filaments, neither do the inter-tubular spaces.

The cement receives its nerve fibrils from the pulp through the anastomosing tubuli and not the pericementum.

In all other respects cement resembles true bone, with this difference: in the cement there is no Haversian canals in its normal condition.

